# Gestational Exposure to Air Pollution Alters Cortical Volume, Microglial Morphology, and Microglia-Neuron Interactions in a Sex-Specific Manner

**DOI:** 10.3389/fnsyn.2017.00010

**Published:** 2017-05-31

**Authors:** Jessica L. Bolton, Steven Marinero, Tania Hassanzadeh, Divya Natesan, Dominic Le, Christine Belliveau, S. N. Mason, Richard L. Auten, Staci D. Bilbo

**Affiliations:** ^1^Department of Psychology and Neuroscience, Duke University, DurhamNC, United States; ^2^Department of Neurobiology, Duke University Medical Center, DurhamNC, United States; ^3^Department of Pediatrics, Division of Neonatal Medicine, Duke University Medical Center, DurhamNC, United States; ^4^Department of Pediatrics and Program in Neuroscience, Lurie Center for Autism, Harvard Medical School, Massachusetts General Hospital for Children, BostonMA, United States

**Keywords:** microglia, air pollution, microglia-neuron interactions, cortical volume, brain development

## Abstract

Microglia are the resident immune cells of the brain, important for normal neural development in addition to host defense in response to inflammatory stimuli. Air pollution is one of the most pervasive and harmful environmental toxicants in the modern world, and several large scale epidemiological studies have recently linked prenatal air pollution exposure with an increased risk of neurodevelopmental disorders such as autism spectrum disorder (ASD). Diesel exhaust particles (DEP) are a primary toxic component of air pollution, and markedly activate microglia *in vitro* and *in vivo* in adult rodents. We have demonstrated that *prenatal* exposure to DEP in mice, i.e., to the pregnant dams throughout gestation, results in a persistent vulnerability to behavioral deficits in adult offspring, especially in males, which is intriguing given the greater incidence of ASD in males to females (∼4:1). Moreover, there is a striking upregulation of toll-like receptor (TLR) 4 gene expression within the brains of the same mice, and this expression is primarily in microglia. Here we explored the impact of gestational exposure to DEP or vehicle on microglial morphology in the developing brains of male and female mice. DEP exposure increased inflammatory cytokine protein and altered the morphology of microglia, consistent with activation or a delay in maturation, only within the embryonic brains of male mice; and these effects were dependent on TLR4. DEP exposure also increased cortical volume at embryonic day (E)18, which switched to decreased volume by post-natal day (P)30 in males, suggesting an impact on the developing neural stem cell niche. Consistent with this hypothesis, we found increased microglial-neuronal interactions in male offspring that received DEP compared to all other groups. Taken together, these data suggest a mechanism by which prenatal exposure to environmental toxins may affect microglial development and long-term function, and thereby contribute to the risk of neurodevelopmental disorders.

## Introduction

Microglia are the resident immune cells of the brain, deriving from primitive macrophage precursor cells in the yolk sac that migrate into the brain beginning on embryonic day (E) 8.5 (Ginhoux et al., 2010). From this early age, microglia play increasingly well-defined roles in normal brain development and homeostasis, beyond host defense, including activity-dependent synapse elimination and the phagocytosis of surplus neural precursor cells (Schafer et al., 2012; Cunningham et al., 2013). Thus, even small perturbations of microglial function early in life can have important consequences for neural development, and for the risk of neurological disorders throughout the lifespan (reviewed in Bilbo and Schwarz, 2012). For instance, genetic ablation of the microglial chemokine receptor CX3CR1 in mice transiently reduces the number of microglia during development, but persistently dysregulates brain connectivity and alters social behavior and communication, which has important relevance for disorders like autism spectrum disorder (ASD) (Paolicelli et al., 2011; Zhan et al., 2014).

The heterogeneous clinical and biological phenotypes observed in ASD strongly suggest that in genetically susceptible individuals, environmental risk factors also combine or synergize to create a tipping or threshold point for dysfunction. Though the mechanisms underlying these associations are likely diverse, many environmental factors or toxins impact immune or inflammatory pathways in the body (Carter and Blizard, 2016). A role for immunological involvement in a significant subtype of ASD has been hypothesized for some time (Money et al., 1971), and results from neuroimaging studies utilizing PET/MRI have identified putative “inflammation” in the brains of some subjects with ASD compared to control subjects (Pardo et al., 2005; Vargas et al., 2005; Suzuki et al., 2013). Similarly, post-mortem studies show that microglia are morphologically altered, again in a subset of individuals, a phenotypic shift often associated with “activation,” though the mechanisms remain largely unclear (Pardo et al., 2005; Vargas et al., 2005; Morgan et al., 2010; Lee et al., 2017).

Air pollution is one of the most pervasive and harmful environmental toxicants in the modern world, and several large-scale epidemiological studies have recently linked prenatal air pollution exposure with an increased risk of ASD (Volk et al., 2011, 2013; Roberts et al., 2013; Raz et al., 2014; Rossignol et al., 2014). Roadway exposures account for the majority of air pollution in the environment, of which diesel exhaust particles (DEP) are a primary toxic component (Inoue et al., 2006a,b,c; Hartz et al., 2008; Block and Calderon-Garciduenas, 2009). DEP markedly activates microglia *in vitro* and *in vivo* in adult rodents (Block et al., 2007, 2012; Block and Calderon-Garciduenas, 2009; Costa et al., 2015). We have demonstrated that *prenatal* exposure to DEP in mice, i.e., to the pregnant dams throughout gestation, results in a persistent vulnerability to behavioral deficits in adult offspring, especially in males (Bolton et al., 2012, 2013, 2014), which is intriguing given the greater incidence of ASD in males to females (∼4:1). Moreover, there is a striking upregulation of toll-like receptor (TLR) 4 gene expression within the brains of the same mice, and this expression is primarily in microglia (Bolton et al., 2013). The impact of DEP on the inflammatory response in the body, e.g., within the lung, is dependent on TLR4 (Inoue et al., 2006b), an innate immune pattern recognition receptor classically defined for its recognition of pathogen-associated molecular patterns (PAMPs), e.g., lipopolysaccharides (LPS) on bacteria. There is growing evidence of TLR4 recognition of putative “danger” associated molecular patterns (DAMPs) as well, including hyaluronic acid, high mobility group box (HMGB)1, heat shock proteins, and other markers of cellular distress or damage, in addition to an increasingly wide array of environmental toxicants that are hypothesized to directly or indirectly act as ligands (Nazeen et al., 2016).

Here we explored the impact of gestational exposure to DEP or vehicle (VEH) on microglial development and activation in the brains of male and female mice. We tested the hypothesis that DEP-induced activation of microglia is dependent on TLR4 by assessing TLR4-competent vs. knockout male and female littermates. DEP exposure increased inflammatory cytokine protein and altered the morphology of microglia, consistent with activation or a delay in maturation, only within the embryonic brains of male mice; and these effects were dependent on TLR4. DEP exposure also induced gross changes in cortical volume in males: increased volume at embryonic day (E)18 that switched to decreased volume by post-natal day (P)30, suggesting an impact on the developing neural stem cell niche. Consistent with this hypothesis, we found increased microglial-neuronal interactions in male offspring that received DEP compared to all other groups. Taken together, these data suggest a mechanism by which prenatal exposure to environmental toxins may affect microglial development and long-term function in a sexually dimorphic manner, and thereby contribute to the risk of neurodevelopmental disorders.

## Materials and Methods

### Experiment 1: E18 Time Point

#### Animals

Adult male TLR4-deficient (TLR4^-/-^) and female TLR4-heterozygous (TLR4^+/-^; C57BL/6 background) mice were housed in individually ventilated, microisolator polypropylene cages with specialized bedding (Alpha-Dri; Shepherd Specialty Papers, Milford, NJ, United States; used to avoid exposure to potentially confounding antigens that can be found in typical bedding) and given *ad libitum* access to food (PicoLab Mouse Diet 5058, Lab-Diet, Philadelphia, PA, United States) and filtered water. The colony was maintained at 22°C on a 12:12-h light-dark cycle (lights on at 7 AM). Following acclimation to laboratory conditions, males were placed with 2 females each for breeding, for a maximum of 2 weeks. TLR4^-/-^ males were mated with TLR4^+/-^ females to produce litters of approximately 50% for each genotype. Females were examined twice daily for evidence of a vaginal plug [confirmation of successful mating, considered to be E0], at which point they were separated from the male and caged with 1–4 other successfully mated females. All experiments were conducted with protocols approved by the Duke University Animal Care and Use Committee.

#### Diesel Exhaust Particle (DEP) Exposures

Beginning on the morning of E2, time-mated females were treated with DEP delivered by oropharyngeal aspiration [the method and detailed analysis of DEP have been previously described (Bolton et al., 2013)]. Females received 50 μg DEP suspended in 50 μl vehicle (DEP group, *n* = 9) or 50 μl vehicle (VEH group, *n* = 7) on E2, E5, E8, E12, and E16.

#### Tissue Collection

Fetal brains were collected at E18. Fetuses were sexed and their *Tlr4* genotype determined by PCR analysis of the *Sry* and *Tlr4* genes from tail samples. At the time of tissue collection, pregnant mice were deeply anesthetized with sodium pentobarbital, and fetuses were removed by hysterotomy. For half of each litter (randomly chosen), each fetus’s whole brain was removed and weighed, before being placed into a 1.5-ml microcentrifuge tube, snap-frozen in liquid nitrogen, and stored at -80°C until processing. For the other half of each litter, each fetus’s whole head was removed and placed in 1.5-ml microcentrifuge tubes filled with 10% formalin. Twenty-four hours later, heads were transferred into 0.1% sodium azide for long-term storage at 4°C. Three days prior to slicing, heads were submerged in a 10% gelatin solution. After the gelatin solidified, the gelatin-submerged heads were transferred to cold 4% paraformaldehyde for 24 h before being transferred into 30% sucrose for 48 h prior to freezing and slicing.

#### Genotyping

DNA extraction of tail snips was performed by heating the samples for 1 h at 95°C in a basic solution (25 mM NaOH, 0.2 mM EDTA), then adding an acidic solution (40 mM Tris HCl) to neutralize the pH. Each sample was centrifuged at 400 × *g* for 10 min at 4°C, followed by collection of the supernatant. Subsequently, PCR amplification was performed, which consisted of heating samples at 94°C for 3 min, followed by 34 cycles of 94°C for 20 s, 67.5–64.5°C for 30 s (the first 6 cycles comprised a touchdown from 67.5 to 64.5°C, in half-degree increments), and 72°C for 40 s, and finished at 72°C for 50 s. The PCR reaction consisted of 1 μl of sample and 24 μl of BIOLASE PCR mix (Bioline USA Inc., Taunton, MA, United States) containing forward (5′-TGGGCTGGACTAGGGAGGTCC-3′) and reverse (5′-TGCTGGGCCAACTTGTGCCT-3′) SRY primers and 3 TLR4 primers: forward (5′- CTG ACG AAC CTA GTA CAT GTG GA), reverse (5′- ACC TCT TAG AGT CAG TTC ATG GA), and Neo-rrt1 (5′- TGG CGG ACC GCT ATC AGG AC). TLR4^+/-^ fetuses were identified by the appearance of 2 bands, one of 280 bp and one of 186 bp, whereas TLR4^-/-^ fetuses have one band of 280 bp. The Y chromosome-specific product expected from the amplification of male DNA was 431 bp. Products were resolved on a 1.7% agarose gel stained with 5% GelStar Nucleic Acid Stain (Cambrex Bio Science Rockland, Inc., Rockland, ME, United States).

#### Cytokine Protein Measurements

Commercial ELISA kits (R&D Systems, Minneapolis, MN) were used to measure interleukin (IL)-1β, a proinflammatory cytokine, and IL-10, an anti-inflammatory cytokine, in fetal brain homogenates normalized to total protein (200 μg/well) and lipid-depleted [*n* = 5–8/sex/genotype/treatment (Bolton et al., 2013)]. These cytokines were selected due to their important role in microglial function, brain development, and behavior (Williamson et al., 2011; Yirmiya and Goshen, 2011).

#### Iba1 Immunohistochemistry

Gelatin-blocked brains were sectioned exhaustively in a 1:5 series at 14 μm on a Leica cryostat at 20°C and thaw-mounted directly onto Superfrost+ Micro Slides (VWR), where they were allowed to dry before being stored at 4°C. The ionized calcium-binding adaptor molecule (Iba1) protein was selected for staining because it is specific to macrophages, including microglia within the brain parenchyma, its expression is constitutive, and it labels the entire cell body including processes, thus allowing a detailed assessment of morphology. Slides were washed with phosphate buffered saline (PBS) and incubated for 1 h in PBS with 1% H_2_O_2_, 10% normal goat serum, and 0.9% Triton X to quench endogenous peroxidase, block, and permeabilize, respectively. Slides were then incubated with 200 μL of primary antibody (rabbit anti-Iba1, 1:500; Wako Chemicals, Richmond, VA, United States) overnight at room temperature. On the next day, slides were washed and incubated with a biotinylated secondary antibody (goat anti-rabbit IgG, 1:200; Jackson ImmunoResearch Laboratories, West Grove, PA, United States) for 2 h at room temperature. Slides were washed, and immunostaining was identified by the streptavidin/horseradish peroxidase technique (Vectastain ABC kit; Vector Laboratories, Burlingame, CA, United States) with diaminobenzidine as the chromagen. Afterward, slides were dehydrated and coverslipped with Permount (Fisher Scientific, Pittsburgh, PA, United States).

#### Unbiased Stereology

Iba1-labeled cells were counted using the optical fractionator method within Stereo Investigator software (Microbrightfield Inc., Williston, VT, United States) (Mouton, 2002; Glaser et al., 2007; Bland et al., 2009). For analysis, we set an optical dissector height of 5 μm with a 1-μm guard zone on top and bottom, and counted stained cells within each frame using a 100X oil objective lens. Cells were only counted if the entire, rounded cell body was visible (average diameter = 15–25 μm) and the stain appeared uniformly dark and apparent throughout the cell, to avoid counting a cell fragment.

We used an exhaustive 50 μm by 50 μm counting frame to count cells throughout each section for each section counted. For each animal, we analyzed every section throughout the parietal cortex (PCX) and hippocampus [dentate gyrus (DG), CA3, and CA1]. Nine sections of the PCX were analyzed for every animal, 7 per DG, 4 per CA3, and 6 per CA1. For each section examined, the area was calculated using Stereo Investigator software based on the boundaries of the contour tracings (Supplementary Figures 1A,B). Regional volume estimates were obtained by summing the areas given by the Cavalieri estimator for each section, and then multiplying this value by the product of the pre-histology thickness of each section (14 μm) and the number of sections examined.

**FIGURE 1:**
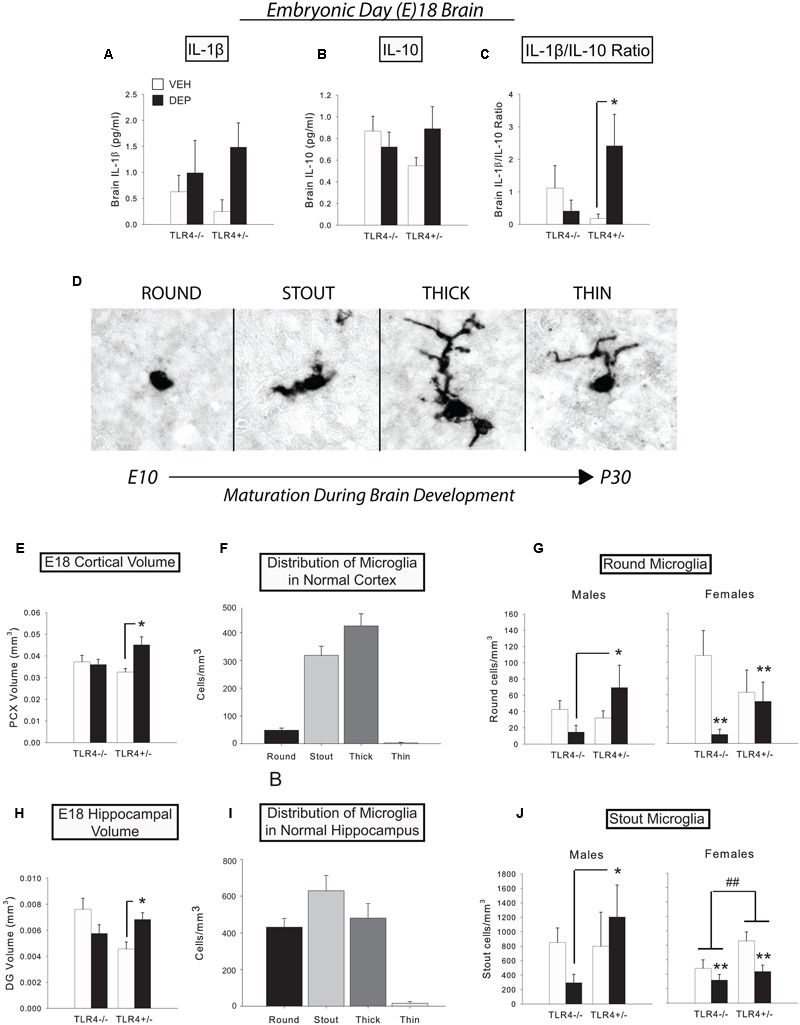
**Prenatal DEP exposure alters microglial morphology and gross regional volumes in the E18 fetal brain. (A)** Levels of IL-1β tended to be higher in E18 brains due to prenatal DEP exposure. **(B)** Levels of IL-10 did not significantly differ in the E18 brain. **(C)** The proinflammatory bias (IL-1β/IL-10 ratio) was significantly greater in DEP TLR4^+/-^ E18 brains than in VEH TLR4^+/-^ E18 brains. **(D)** The four primary microglial morphological states on E18. During development, microglia possess round/amoeboid morphology initially and gradually progress to a morphology with thin, ramified processes. Photos were taken of Iba1-labeled microglia in the mouse CA1 at the 100X objective. **(E)** DEP TLR4^+/-^ animals possess a significantly larger PCX volume than VEH TLR4^+/-^ animals and DEP TLR4^-/-^ animals at E18. **(F)** Normal distribution of microglial morphological states in E18 PCX. **(G)** DEP TLR4^+/-^ males have significantly more round microglia in the PCX than DEP TLR4^-/-^ males. In contrast, VEH females have significantly more round microglia than DEP females in the PCX at E18. **(H)** DEP TLR4^+/-^ animals possess a significantly greater DG volume at E18 than VEH TLR4^+/-^ animals. **(I)** Normal distribution of microglial morphological states in E18 DG. **(J)** DEP TLR4^+/-^ males have significantly more stout microglia than DEP TLR4^-/-^ males in the E18 DG, whereas DEP females have significantly fewer stout microglia overall than VEH females. In addition, TLR4^+/-^ females have overall more stout microglia than TLR4^-/-^ females. Data are mean ± SEM. For A-C, *n* = 6–13/group (note that sexes are combined here). For E and H, *n* = 8–9/group (note that sexes are combined here). For **(F,I)** *n* = 33 animals/morphology. For **(G,J)** *n* = 3–5/group/sex. ^∗^*p* < 0.05 vs. VEH TLR4^+/-^ or DEP TLR4^-/-^; ^##^*p* < 0.05, TLR4^+/-^ vs. TLR4^-/-^; ^∗∗^*p* < 0.05, DEP vs. VEH.

Iba1-positive cells were classified into 4 morphological types based on their cell shape and the configuration of their processes (Wu et al., 1992, 1993; Kreutzberg, 1996; Gomez-Gonzalez and Escobar, 2010). These four cell types consisted of round/amoeboid microglia, microglia with stout processes, microglia with thicker, longer processes, and microglia with thinner, more ramified processes (see **Figure 1D**). The number of subjects analyzed varied per region and group, *n* = 3–5/group/sex for the PCX, DG, CA1, and CA3.

#### Data Analysis

All data were analyzed with SigmaStat statistical software (Systat Software Inc., San Jose, CA, United States). Three-way (DEP X Genotype X Sex) ANOVAs were performed first, and following an interaction with Sex, two-way (DEP X Genotype) ANOVAs within each sex were used to assess differences in regional volume (mm^3^), the total number of cells/mm^3^, and the number of cells of the 4 different morphologies for microglia/mm^3^. Following significant interactions, *post hoc* comparisons (Fisher’s LSD) were performed to further distinguish between groups. Significance was generally assumed at *p* < 0.05; however, higher-order interactions at *p* ≤ 0.1 were used to direct the subdivision of data for further analysis (Snedecor and Cochran, 1967). These interactions were only reported if subsequent lower-order ANOVAs and/or *post hoc* tests achieved significance at *p* < 0.05 (Slotkin and Seidler, 2015).

### Experiment 2: P30 Time Point

#### Animals

Adult male and female C57BL/6 mice were obtained from Charles River Laboratories (Raleigh, NC, United States) and housed in individually ventilated, microisolator polypropylene cages with specialized bedding (Alpha-Dri) and *ad libitum* food (PicoLab Mouse Diet 5058; Lab-Diet, Philadelphia, PA, United States) and filtered water. The colony was maintained at 22°C on a 12:12-h light–dark cycle (lights off at 9 AM). Following acclimation to laboratory conditions for 1 week, males were paired with 2 females each for timed mating, for a maximum of 2 weeks. Females were examined twice daily for evidence of a vaginal plug [confirmation of successful mating, considered to be embryonic day (E)0], at which point they were separated from the male and housed with another pregnant female. All experiments were conducted with protocols approved by the Duke University Animal Care and Use Committee.

#### DEP Exposures

Female DEP treatment was carried out as described above. Females received 50 μg DEP suspended in 50 μl vehicle (DEP group; *n* = 5 dams) or vehicle alone (VEH group; *n* = 7 dams) every 3 days E2-E17 for a total of 6 doses, as a model of intermittent exposure. Following the final exposure on E17, females were allowed to give birth normally and their offspring remained unmanipulated until P30.

#### P30 Injections

P30 offspring (*n* = 6–8/group/sex) received an i.p. injection of either sterile saline or 165 μg/kg LPS derived from Escherichia coli (serotype 0111:B4; Sigma, St. Louis, MO, United States) between 8 and 10 AM, and brain tissues were collected 2 h later. This dose and time point were selected based on previous studies (Godbout et al., 2005) and initial pilot experiments from our lab demonstrating mild sickness behavior and a robust but submaximal IL-1β response under these conditions in the brains of control mice (unpublished data).

#### Tissue Collection

Mice were deeply anesthetized with a ketamine/xylazine cocktail (430 mg/kg ketamine; 65 mg/kg xylazine i.p.). Immediately afterward, mice were transcardially perfused with ice-cold saline for 2 min. Whole brains were rapidly extracted, and half the brain was post-fixed by 4 successive changes of fresh 4% paraformaldehyde daily, and then stored in 0.1% sodium azide in PBS at 4°C until cryosectioning for histological analyses. All tissue collection occurred during the dark cycle between 10 AM and 1 PM.

#### Iba1 Immunohistochemistry

Fixed half-brains were cryoprotected in 30% sucrose plus 0.1% sodium azide for 3 days prior to slicing, then quickly frozen in -70°C isopentane and sliced coronally from the anterior hypothalamus through the ventral hippocampus in a 1:6 series at 40 μm in a -20°C cryostat. Floating sections were stored in a 0.1% sodium azide solution until immunohistochemistry was performed. All sections were stained at the same time to minimize differences in antibody preparation and binding across days.

As in Experiment 1, Iba1 staining was performed. Free-floating sections were washed with PBS, incubated in 50% methanol for 30 min to quench blood vessels, and then washed in PBS again. They were then incubated for 1 h in PBS with 0.03% H_2_O_2_, 5% normal goat serum, and 0.3% Triton X to quench endogenous peroxidase, block, and permeabilize, respectively. Sections were incubated with primary antibody (rabbit anti-Iba1, 1:1,000; Wako Chemicals, Richmond, VA, United States) overnight at room temperature on an orbital shaker. On day 2, sections were washed and incubated with a biotinylated secondary antibody (goat anti-rabbit IgG, 1:200; Vector Laboratories, West Grove, PA, United States) for 2 h at room temperature. Sections were washed, and immunostaining was identified by the streptavidin/HRP technique (Vectastain ABC kit; Vector Laboratories, Burlingame, CA, United States) with diaminobenzidine (DAB) as the chromagen. Sections were mounted on gelatinized slides, dehydrated, and coverslipped with Permount (Fisher Scientific, Pittsburgh, PA, United States).

#### Unbiased Stereology

**FIGURE 2:**
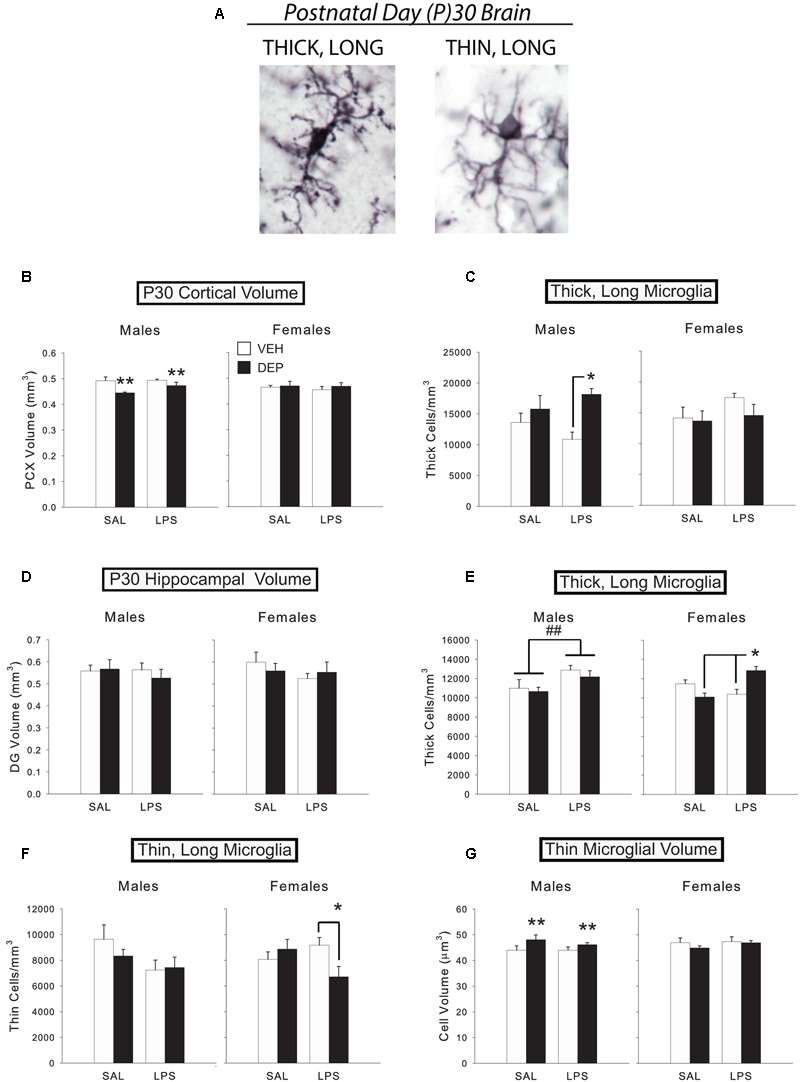
**Prenatal DEP exposure alters microglial morphology and cortical volume in the P30 juvenile brain. (A)** The two primary microglial morphological states in the P30 brain. Representative pictures of microglia with thick, long processes (left) and thin, long processes (right) in the mouse parietal cortex on P30. Photos were taken of Iba1-labeled microglia at 100X. **(B)** DEP males possess a significantly smaller PCX volume at P30 than VEH males, whereas females do not differ. **(C)** DEP/LPS males have significantly more thick, long microglia in the PCX at P30 than do VEH/LPS males, whereas females do not differ. **(D)** No significant differences in DG volume were detected. **(E)** LPS males had significantly more thick, long microglia in the DG at P30 than SAL males. DEP/LPS females had significantly more thick, long microglia than VEH/LPS and DEP/SAL females. **(F)** LPS males tended to have fewer thin, long microglia than SAL males in the P30 DG, whereas DEP/LPS females had significantly fewer thin, long microglia than VEH/LPS and DEP/SAL females. **(G)** DEP males have thin, long microglia with larger soma volumes in the DG at P30 than do VEH males, whereas females do not differ. Data are mean ± SEM, *n* = 5–7/group/sex. ^∗∗^*p* = 0.05, DEP vs. VEH; ^∗^*p* < 0.05 vs. VEH/LPS or DEP/SAL; ^##^*p* < 0.05, LPS vs. SAL.

Iba1-labeled cells were quantified using Stereo Investigator software as described previously. Iba-1 positive cells in the P30 brain were classified as either “thick, long,” with broad processes, or “thin, long,” with ramified processes, which encompass the large majority of cell types at this age (**Figure 2A**). Unbiased stereology (Gundersen and Jensen, 1987) was used to estimate total numbers of Iba1-labeled cells and total numbers of each morphology. Microglial cell volumes were estimated using five rays of independent isotropic probes within the “Nucleator” function of Stereo Investigator software. We analyzed five sections through the PCX and DG (Supplementary Figure 1C) for each animal. For each section examined, the area was calculated by the Stereo Investigator software based on the boundaries of the contour tracings. Regional volume estimates were obtained by summing the areas given by the Cavalieri estimator for each section, and then multiplying this value by the product of the pre-histology thickness of each section (40 μm) and the number of sections examined. The estimated total number of microglia per region was obtained using Stereo Investigator’s Estimated Total by Mean Measured Thickness function, which bases its calculation on section thickness, series value, and number of measurements. These values were normalized by regional volume. The number of subjects analyzed per group was 5–7/group/sex for all regions analyzed.

#### Iba1-NeuN Fluorescence Immunohistochemistry

The brains of SAL-injected animals were processed to interrogate microglial-neuronal interactions. Microglia were detected with a rabbit polyclonal antibody against Iba1 (#019-19741; Wako Chemicals). Neurons were detected with a mouse monoclonal antibody against NeuN, clone A60 (MAB377; Millipore). The protocol for all antibodies was similar to that described above, and PBS was used throughout to wash the sections. Sections were blocked with 5% normal donkey serum (NDS) with 0.3% Triton-X in PBS at room temperature for 1 h. Primary antibodies were diluted in a solution of 5% NDS and 0.3% Triton-X in PBS to yield dilutions of 1:1,000 primary antibody for both Iba1 and NeuN. Sections were incubated overnight at 4°C. For fluorescent signal detection, the following Alexa Fluor conjugated secondary antibodies were used: Donkey anti-mouse-488, donkey anti-rabbit-568 (1:500, both purchased from Thermo Fisher Scientific). Sections were incubated in the dark for 2 h at room temperature. Labeled sections were mounted onto glass slides and cover slipped with fluorescent mounting medium (Vector laboratories).

#### Confocal Microscopy

Immuno-labeled PCX sections were viewed on a Leica SP8 upright microscope equipped with HC Plan-Apochromat 40× (oil numerical aperture, NA: 1.3) or 100× (oil DIC; NA 1.4) objectives and attached to a spectral confocal laser system with Argon/2 (458, 488, and 514 nm), 561 nm Diode, and HeNe 633 nm. The tissue was scanned with 488-nm and 561-nm laser lines to detect the corresponding Alexa fluorophores. High-resolution images (1024 × 1024 pixels) of optical sections (*z*-slices) were captured using sequential line (average of 3) scanning with 0.3-μm *z*-steps.

#### Quantification of Immunohistochemistry

**FIGURE 3:**
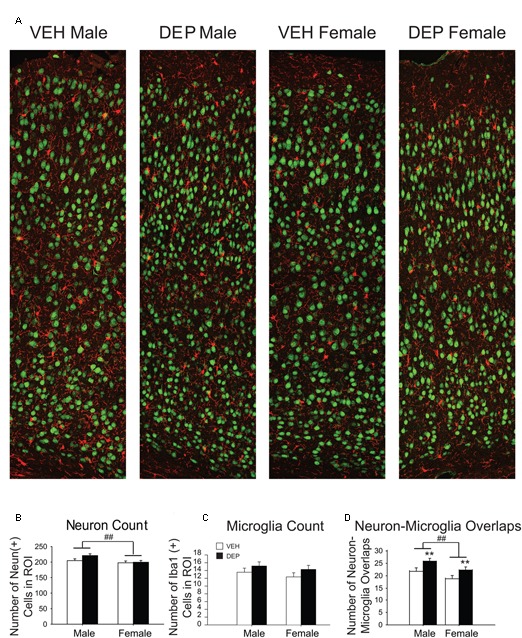
**Prenatal DEP exposure alters microglia-neuron interaction frequency but not neuron or microglia number in the parietal cortex of P30 offspring. (A)** Representative panoramic images of microglia (Iba1, red) and neurons (NeuN, green) in the parietal cortex of P30 offspring taken at 40× magnification and stitched together with 30% overlap. **(B)** Neuron Quantification. Males have significantly more NeuN+ cells than females. **(C)** Microglia Quantification. The numbers of Iba1+ cells do not differ by Sex, DEP exposure or the interaction of the two. **(D)** Microglia-Neuron Interaction Quantification. Males have significantly more overlaps between microglia and neurons than females, and DEP-treated animals have significantly more overlaps than those treated with VEH. Bar graphs represent mean values ± SEM (error bars); *n* = 6 mice/group, average of 3 brain sections per mouse. ^##^*p* < 0.05, males vs. females; ^∗∗^*p* < 0.05, DEP vs. VEH.

Three independent 40 μm-thick coronal brain sections containing the PCX (bregma -1.455 to -2.355) were imaged per mouse in 5-μm-thick confocal scans (optical section depth of 0.333 μm, 15 sections/scan) 40× magnification. A series of images were collected from the pia to the corpus callosum with 30% overlap (average of 4 images). ImageJ was used to create maximum projections and subtract background. Following the background subtraction, each image series was merged together using Adobe Photoshop until the image series of each section was cohesively stitched together with 30% overlap (**Figure 3A**).

Quantification of NeuN+ cells was completed using a previously published protocol (McKinstry et al., 2014). The stitched images of the cortices were divided into 10 equal parts (identical dimensions in all images) spanning the distance between the pia and the corpus callosum. The number of NeuN+ neurons and Iba1-NeuN/ microglia-neuron cell body overlaps were counted using the Cell Counter Plugin for ImageJ (Schneider et al., 2012) in the tiled images. Microglia-neuron cell body overlaps were identified based on the appearance of cell body-cell body contact as determined by co-localization (yellow) of Iba1 and NeuN within the entire brain section (*n* = 6 mice/group; 3 brain sections/mouse).

#### Three-Dimensional (3D) Reconstruction of Microglia

Confocal z-scans of Iba1-positive microglia were acquired through a 100× objective (oil DIC; NA 1.4) in 0.3-μm *z*-steps. Image series were saved in a.lif format and ImageJ was used to perform background subtraction, smoothing, and thresholding. Imaris software (Bitplane) was used to perform 3D reconstruction and surface renderings of microglia, neurons, and the overlap between them (Schafer et al., 2012, 2014). The 3D renderings of the microglia-neuron interactions were then observed to verify that they met criteria to be included (detailed below). Those that passed inspection were cleared of extraneous microglia and neurons so that only the microglia and neuron directly involved in cell body-cell body contact remained. These surface renderings were used to quantify the volume of microglia as well as the volume of overlap between microglia and neurons.

#### Criteria for Microglia-Neuron Interaction Rendering

**FIGURE 4:**
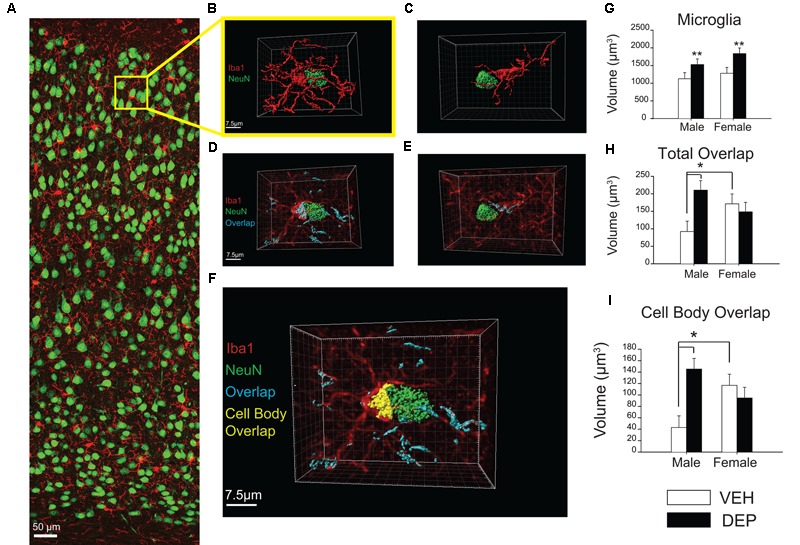
**Increased overlap between microglia and neurons in juvenile offspring prenatally exposed to DEP. (A)** Representative panoramic image of microglia (Iba1, red) and neurons (NeuN, green) in the parietal cortex of a P30 DEP-exposed female taken at 40× magnification and stitched together with 30% overlap. **(B)** Example 3D surface rendering of microglia-neuron apposition, imaged by confocal microscopy at 100× magnification and reconstructed following a previously published protocol (Schafer et al., 2012, 2014). Neurons and microglia not involved in the microglia-neuron interaction were removed for clarity. **(C)** Example of microglia-neuron apposition that was excluded from analysis post-reconstruction due to lack of direct cell body contact. **(D,E)** 3D surface renderings of neurons (NeuN, green) and microglia-neuron overlapping regions (cyan). In these images, 3D reconstructed microglia appear in translucent red to allow visualization of overlapping material within microglia. Note: neurons not directly contacting the microglial cell body were removed for clarity but overlapping regions between microglial processes and these extraneous neurons were quantified as part of total overlap between microglia and neurons. **(F)** 3D reconstruction of microglia-neuron apposition including total overlapping material (cyan) and cell-body overlap (yellow). **(G)** Quantification of microglial cell volume. Analysis of reconstructed microglial volume revealed DEP-exposed offspring have larger microglia volumes than those exposed to VEH. **(H)** Quantification of total microglia-neuron overlap. Total overlap includes overlapping material between microglial processes and any surroundings neurons, as well as cell body-cell body overlap. Analysis of the total volume of Iba1-NeuN overlap revealed a significant Sex X DEP interaction, which is driven by a larger overlap volume in DEP males vs. VEH males, as well as a larger overlap volume in VEH females vs. VEH males. **(I)** Quantification of cell body overlap. Cell body overlap is restricted to overlap between one microglia and one neuron, and just between the cell bodies of these two cells. Analysis of cell body overlap revealed a significant Sex X DEP interaction, which we determined to be due to a larger volume of cell body overlap in DEP males vs. VEH males, as well as in VEH females vs. VEH males. Bar graphs represent mean values ± SEM (error bars); *n* = 3 mice/group, 3–5 microglia-neuron renderings per mouse. The data were log-transformed for analysis due to unequal variance, but are displayed as raw data in the figures. Grid line increments = 2.5 μm. ^##^*p* < 0.05, males vs. females; ^∗∗^*p* < 0.05, DEP vs. VEH; ^∗^*p* < 0.05, vs. VEH male.

One section containing microglia-neuron appositions identified in our stitched 40× images was taken from three different brains for each group (3 mice/group; 3–5 microglia-neuron interaction renderings per mouse). Using confocal microscopy, z-stacks of approximately 40-μm were collected in 0.3-μm *z*-steps. From these *z*-stacks, microglia were chosen for analysis based on the following criteria: (1) 3D renderings contained microglia-neuron cell body contact, (2) full microglial cell bodies that did not touch the *z*-stack perimeter, and (3) processes were only included if they were clearly attached to the microglia of interest. A total of 101 microglia were collected in this way, 45 of which met criteria and were included. Thirty-nine were excluded for not meeting criteria (**Figure 4C** for example) and 17 were not rendered because our target n was reached.

#### Data Analysis

All data were analyzed with SigmaStat statistical software (Systat Software Inc., San Jose, CA, United States). Three-way (DEP X LPS X Sex) ANOVAs were performed first, and following an interaction with Sex, two-way (DEP X LPS) ANOVAs within each sex were used to assess differences in regional volume (mm^3^), average cell volume (μm^3^), the total number of cells/mm^3^, and the number of cells (of the 2 different morphologies)/mm^3^. Following interactions, *post hoc* comparisons (Fisher’s LSD) were performed to further distinguish between groups. For neuron counts and microglial-neuron overlap, two-way (DEP X Sex) ANOVAs were performed to assess differences in the number of neurons, number of microglia-neuron interactions, microglial volume (μm^3^), and overlap volume (μm^3^). Interactions were followed up with Fisher’s LSD *post hoc* tests. The 3D reconstruction data were log-transformed for analysis due to unequal variance, but are displayed as raw data in the figures. Significance was generally assumed at *p* < 0.05; however, higher-order interactions at *p* ≤ 0.1 were used to direct the subdivision of data for further analysis (Snedecor and Cochran, 1967). These interactions were only reported if subsequent lower-order ANOVAs and/or *post hoc* tests achieved significance at *p* < 0.05 (Slotkin and Seidler, 2015).

## Results

### Experiment 1: E18 Time Point

#### Fetal Brain Cytokines

We measured the levels of proinflammatory IL-1β and anti-inflammatory IL-10 in fetal brains at E18 to determine if prenatal DEP exposure induces a TLR4-dependent cytokine response in the fetal brain. Prenatal DEP exposure tended to increase IL-1β (*p* = 0.07; **Figure 1A**), whereas there were no differences in IL-10 levels in the fetal brains (**Figure 1B**). We next analyzed the IL-1β/IL-10 ratio and found that DEP TLR4^+/-^ fetal brains exhibited a significantly greater proinflammatory bias than did VEH TLR4^+/-^ fetal brains [DEP X Genotype interaction, *F*(1,22) = 3.14, *p* = 0.09; *post hoc, p* < 0.05], whereas TLR4^-/-^ brains did not differ due to prenatal treatment (**Figure 1C**).

#### Fetal Brain Microglia Counts and Morphological Analysis

To assess whether prenatal DEP exposure altered microglial development, we exhaustively counted microglia and classified them into four morphological types (**Figure 1D**) in the DG, CA1, CA3, and PCX. We also measured the volume of each brain region to determine if there were any gross changes in brain structural development due to prenatal DEP exposure.

##### Parietal cortex

Overall volume of the PCX was significantly increased by DEP exposure in TLR4^+/-^ fetuses compared to VEH-treated TLR4^+/-^ fetuses, whereas TLR4^-/-^ fetuses did not differ by treatment [significant DEP X Genotype interaction, *F*(1,26) = 5.34, *p* < 0.05; *post hoc, p* < 0.05; **Figure 1E**]. No significant differences were detected in overall microglial density. However, round microglia were significantly altered by the interaction of DEP and TLR4 genotype [DEP X Genotype interaction, *F*(1,27) = 5.42, *p* < 0.05; Sex X DEP interaction, *F*(1,27) = 3.02, *p* = 0.09]. Specifically, DEP TLR4^+/-^ males had more round microglia than DEP TLR4^-/-^ males [*F*(1,13) = 6.07, *p* < 0.05; *post hoc, p* < 0.05; **Figure 1G**). In contrast, DEP females exhibited the opposite pattern, showing a decrease in round microglia relative to VEH females [significant main effect of DEP, *F*(1,13) = 5.03, *p* < 0.05; **Figure 1G**]. No further differences were detected in stout; thick; or thin microglia (normal distribution of types shown in **Figure 1F**).

##### Dentate gyrus

Overall volume of the DG followed a similar pattern to the volume change observed in the PCX, in that volumes were significantly greater in DEP-treated TLR4^+/-^ fetuses than in VEH-treated TLR4^+/-^ fetuses [significant DEP X Genotype interaction, *F*(1,18) = 9.44, *p* < 0.01; *post hoc, p* < 0.05; **Figure 1H**]. No significant differences were detected in total microglial density or round cell density. However, stout cells exhibited a pattern similar to that observed for round cells in the PCX [Sex X DEP X Genotype interaction, *F*(1,19) = 3.88, *p* = 0.06]. Specifically, DEP TLR4^+/-^ males had significantly more stout microglia than DEP TLR4^-/-^ males [DEP X Genotype interaction, *F*(1,9) = 2.77, *p* = 0.1; *post hoc, p* < 0.05; **Figure 1J**]. In contrast, DEP females had fewer stout microglia than VEH females [significant main effect of DEP, *F*(1,9) = 7.49, *p* < 0.05], and TLR4^+/-^ females had more stout microglia than TLR4^-/-^ females [significant main effect of Genotype, *F*(1,9) = 5.37, *p* < 0.05; **Figure 1J**]. Analysis of thick microglia revealed a significant DEP X Genotype interaction [*F*(1,18) = 6.89, *p* < 0.05] that appeared to be driven by females [significant DEP X Genotype interaction, *F*(1,9) = 9.60, *p* < 0.05], due to an increased number of thick microglia in DEP TLR4^-/-^ females compared to VEH TLR4^-/-^ and DEP TLR4^+/-^ (*p* < 0.05; data not shown). No significant differences were detected in thin microglia (normal distribution of types shown in **Figure 1I**).

##### CA3

Overall volume of the CA3 did not differ by Sex, DEP, or Genotype (Supplementary Figure 2A), nor were any differences detected in total microglia density or round cell density. However, stout microglia exhibited a similar pattern as detected in the DG [significant Sex X DEP interaction, *F*(1,16) = 5.03, *p* < 0.05; Sex X DEP X Genotype interaction, *F*(1,16) = 2.44, *p* = 0.1]. Specifically, DEP TLR4^+/-^ males possessed more stout microglia than VEH TLR4^+/-^ and DEP TLR4^-/-^ males [DEP X Genotype interaction, *F*(1,9) = 3.52, *p* = 0.09; *post hoc, p* < 0.05; Supplementary Figure 2B]. Once again, females exhibited the opposite pattern, as DEP females possessed fewer stout cells than VEH females [significant main effect of DEP, *F*(1,7) = 6.88, *p* < 0.05; Supplementary Figure 2B]. Analysis of thick microglia revealed a pattern similar to that of stout cells in males, but it did not reach significance. Thin cells were not different by Sex, DEP, or Genotype.

##### CA1

Overall volume of the CA1 did not differ by Sex, DEP, or Genotype (Supplementary Figure 2C), nor were any differences detected in total microglia density. Analysis of round microglia revealed a significant main effect of Sex [*F*(1,20) = 6.79, *p* < 0.05], with females possessing overall more round microglia than males (data not shown). The pattern of stout cells was similar to that found in the DG and CA3 [significant Sex X DEP X Genotype interaction, *F*(1,20) = 11.29, *p* < 0.005]. DEP TLR4^+/-^ males possessed more stout microglia than DEP TLR4^-/-^ males [DEP X Genotype interaction, *F*(1,10) = 4.82, *p* = 0.05, *post hoc, p* < 0.05; Supplementary Figure 2D]. On the other hand, VEH TLR4^+/-^ females had more stout microglia than DEP TLR4^+/-^ females [significant DEP X Genotype interaction, *F*(1,10) = 7.74, *p* < 0.05, *post hoc, p* < 0.05; Supplementary Figure 2D]. A similar pattern in females for thick cells was present, but it failed to reach significance. No significant differences in thin cells were detected.

### Experiment 2: P30 Time Point

#### P30 Brain Microglia Counts and Morphological Analysis

The results from Experiment 1 demonstrate that prenatal DEP exposure alters microglial morphology in TLR4-competent males, whereas females often exhibit the opposite pattern. In order to determine if prenatal DEP exposure also results in long-term changes in microglial number and/or morphology, in conjunction with the long-term changes in behavior and proinflammatory function that we have observed in previous studies (Bolton et al., 2012, 2013, 2014), we used unbiased stereology to count microglia and classified them into thick, long vs. thin, long processes (the only phenotypes present in the normal adult brain; **Figure 2A** for examples) in the wild-type P30 DG and PCX. Because the number of morphological types in the adult brain are limited, we also measured microglial soma volume to more rigorously assess any changes in morphology. We limited our analyses to the DG of the hippocampus because only the DG possessed a volume change like the PCX at E18, which would suggest potential neuropathology or altered neural-glial interactions in these regions. As at E18, we again measured the volume of each brain region to determine if there were any long-lasting gross changes in brain structure due to prenatal DEP exposure. Half of the animals were also injected with LPS to determine if group differences in function were revealed in response to a second activation of the immune system.

##### Parietal cortex

Analysis of PCX volume revealed a significant Sex X DEP interaction [*F*(1,43) = 6.07, *p* < 0.05], which was due to a smaller volume in DEP males compared to VEH males [significant main effect of DEP, *F*(1,20) = 8.55, *p* < 0.01], whereas females did not differ by treatment (**Figure 2B**). There was no effect of LPS on PCX volume. Analysis of total microglial cell density also revealed a significant Sex X DEP interaction [*F*(1,42) = 5.88, *p* < 0.05], however, *post hoc* tests were not significant (data not shown). Subdividing microglia by morphological type, we discovered that this pattern was mostly driven by thick, long cells, which also exhibited a significant Sex X DEP interaction [*F*(1,42) = 8.16, *p* < 0.01], and a Sex X DEP X LPS interaction [*F*(1,42) = 2.84, *p* = 0.1], whereas thin, long cells did not exhibit any significant differences. Specifically, DEP/LPS males have more thick, long microglia than VEH/LPS males [significant main effect of DEP, *F*(1,20) = 9.00, *p* < 0.01; DEP X LPS interaction, *F*(1,20) = 2.65, *p* = 0.1; *post hoc, p* < 0.05], whereas females exhibit the opposite pattern, although this didn’t reach significance (**Figure 2C**). No significant differences in average microglial cell volume in PCX were identified.

##### Dentate gyrus

Neither overall volume of the DG (**Figure 2D**) nor total microglial cell density differed by Sex, DEP, or LPS. However, subdividing microglia by morphological type revealed a significant Sex X DEP X LPS interaction [*F*(1,40) = 7.17, *p* < 0.05] within thick, long microglia. Follow-up tests that subdivided by sex revealed that LPS-injected males had significantly more thick, long microglia in the DG than SAL-injected males [significant main effect of LPS, *F*(1,21) = 7.24, *p* < 0.05], whereas females exhibited a significant DEP X LPS interaction [*F*(1,19) = 18.53, *p* < 0.001; **Figure 2E**]. Specifically, DEP/LPS females had significantly more thick, long microglia in the DG than VEH/LPS females and DEP/SAL females (*post hoc, p* < 0.005), much like the pattern we observed in males in the PCX. This effect was coupled with a significant Sex X DEP X LPS interaction [*F*(1,40) = 4.76, *p* < 0.05] within thin, long microglia that represented the opposing pattern. Specifically, LPS males tended to have fewer thin, long microglia than SAL-injected males (*p* = 0.06), whereas DEP/LPS females had fewer thin, long microglia than VEH/LPS females [significant DEP X LPS interaction, *F*(1,19) = 5.50, *p* < 0.05; *post hoc, p* < 0.05; **Figure 2F**]. Therefore, it appears that animals maintained the same overall amount of microglia in the DG by converting thin, long microglia to thick, long microglia in response to LPS.

Analysis of average microglial cell volume in the DG revealed a Sex X DEP X LPS interaction [*F*(1,40) = 3.60, *p* = 0.06]. Subdividing by morphological type, we found that thick, long cell volume overall tended to increase in LPS animals (*p* = 0.06; data not shown). On the other hand, analysis of thin, ramified cell volume revealed a significant Sex X DEP interaction [*F*(1,40) = 4.18, *p* < 0.05] that is driven by DEP males having larger soma volumes than VEH males [main effect of DEP, *F*(1,20) = 4.36, *p* = 0.05], whereas female cell volumes do not differ (**Figure 2G**).

##### Quantification of NeuN and Iba1 in P30 parietal cortex

To determine whether the differences in P30 PCX volume were associated with changes in cell number, NeuN+ and Iba1+ cells were counted (**Figure 3A**). We focused our assessment on neurons and did not include additional cell types that could also impact volume (e.g., astrocytes), as it was beyond the scope of the study. Quantification of NeuN revealed that males had significantly more neurons than females [significant main effect of Sex, *F*(1,20) = 5.94, *p* < 0.05] while no significant differences were found by treatment group (*p* > 0.2; **Figure 3B**). The numbers of Iba1+ cells did not differ significantly by Sex or treatment group (**Figure 3C**).

##### Iba1-NeuN quantification in parietal cortex

During the process of creating representative images of the PCX (**Figure 3A**), a number of unusual cell body-cell body overlaps were observed between microglia and neurons. These interactions were quantified to determine if they were influenced by sex or prenatal exposure to DEP. Males contained significantly more overlaps than females [significant main effect of Sex, *F*(1,20) = 5.75, *p* < 0.05] and DEP-treated animals had significantly more overlaps than VEH [significant main effect of DEP, *F*(1,20) = 6.98, *p* < 0.05] (**Figure 3D**).

##### 3D reconstruction analysis of microglial-neuronal interactions in parietal cortex

To further investigate these microglia-neuron interactions, 3D surface renderings were created of microglia, neurons, and microglia-neuron overlaps (**Figures 4A,B**). Of the microglia that were determined to be in direct contact with neurons (see criteria in methods, **Figures 4C–E**), analysis of reconstructed whole microglial volume revealed that DEP-exposed offspring have larger average microglial volumes than those exposed to VEH [main effect of DEP, *F*(1,41) = 8.51, *p* < 0.01; **Figure 4G**]. Analysis of the volume of the microglial cell body, excluding processes, revealed that DEP-exposed offspring also have larger average cell body volumes than those exposed to VEH [main effect of DEP, *F*(1,41) = 5.14, *p* < 0.05; [Supplementary Figure 3A]. Next, the volume of overlap between microglia and neurons was measured. Overlap volume was quantified in two ways: (1) the total volume of all overlapping material associated with the microglia of interest; this included microglia-neuron overlap at microglia processes as well as overlap between the microglia cell body and neuron cell body and (2) the volume of cell body overlap; this volume was restricted to overlapping material between the microglia cell body and neuron cell body only (**Figure 4F**). Analysis of the total volume of Iba1-NeuN overlap revealed a significant Sex X DEP interaction [*F*(1,41) = 5.39, *p* < 0.05], which was driven by larger overlap volume in DEP males vs. VEH males (*post hoc, p* < 0.05) as well as larger overlap volume in VEH females vs. VEH males (*post hoc, p* < 0.05; **Figure 4H**). Similarly, analysis of the cell body overlap volume revealed a significant Sex X DEP interaction [*F*(1,41) = 8.96, *p* < 0.01] which we determined to be due to a larger volume of cell body overlap in DEP males vs. VEH males (*post hoc, p* < 0.05), as well as in VEH females vs. VEH males (*post hoc, p* < 0.05; **Figure 4I**). Because significant differences were found for microglia volume between groups, we also analyzed the overlap volumes normalized to microglia volume. Analysis of the total Iba1-NeuN overlap volume normalized to microglia volume did not change the above results [significant Sex X DEP interaction; *F*(1,41) = 5.91, *p* < 0.05, which is due to a larger overlap volume in DEP males vs. VEH males (*post hoc, p* < 0.05)] (Supplementary Figure 3B). Analysis of the cell body Iba1-NeuN overlap volume normalized to microglia volume also did not alter the non-normalized findings [significant Sex X DEP interaction, *F*(1,41) = 8.12, *p* < 0.01, which is due to a larger volume of cell body overlap in VEH females vs. VEH males (*post hoc, p* < 0.05] and in DEP males vs. VEH males (*post hoc, p* < 0.05)] (Supplementary Figure 3D). Results followed a similar pattern whether normalizing by whole microglial volume or microglial cell body volume (Supplementary Figures 3C,E).

## Discussion

We demonstrate that prenatal exposure to DEP, one of the most pervasive environmental toxins in the world, significantly alters microglial development in mice, resulting in long-term changes in microglial morphology and neuron-glia interactions, which we hypothesize are linked to the adverse health outcomes we have observed previously using this model. These effects are more pronounced in males than in females, and support previous data demonstrating long-term changes in male brain cytokine levels and behavior following prenatal air pollution exposure (Bolton et al., 2012, 2013, 2014). To our surprise, we also found that prenatal DEP results in gross changes in cortical volume: increased volume at E18 that switches to decreased volume at P30, again specifically in males. These data led us to investigate whether a decrease in neuron number accounts for the decrease in volume by P30. However, we found slightly *increased* (but non-significant) numbers of cortical neurons in DEP-exposed males. Moreover, we found significantly increased microglial-neuronal overlaps in DEP-exposed males at P30, even controlling for the numbers of neurons and microglia, the latter of which also did not significantly differ. That is, on a per-cell basis, microglia showed a much greater degree of physical contact/overlap with neurons in the P30 cortex of males exposed to DEP compared to all other groups.

The current study is the first to directly test and confirm the critical role of TLR4, an innate pattern recognition receptor, in the effects of prenatal air pollution exposure on microglial activation and/or development in the offspring. TLR4 is necessary for systemic inflammatory responses to DEP in adult mice (Inoue et al., 2006b), suggesting that DEP acts as (or elicits) a “danger” signal to activate this receptor, which leads to downstream inflammatory cytokine production. Consistent with this hypothesis is the finding that fetuses exposed to DEP had a greater inflammatory bias (IL-1β/IL-10 ratio) within the E18 brain compared to VEH. We have previously observed marked upregulation of TLR4 expression in the brains of adult male offspring in response to prenatal DEP in combination with either maternal stress during gestation or high-fat diet during adulthood (Bolton et al., 2013, 2014). Moreover, these same males showed greater adverse cognitive (memory impairment), mood (anxiety), and neuroinflammatory outcomes compared to females. The prevalence of autism is four times higher in males than females (Stone et al., 2004), and a growing literature shows males often have a poorer outcome to prenatal complications (Bale and Epperson, 2017). For example, male offspring of asthmatic mothers are more likely to suffer severe complications, such as premature birth or stillbirth, than female offspring in response to an acute asthma exacerbation during pregnancy (Murphy et al., 2003).

Interestingly, we observed TLR4-dependent changes not only in microglial morphology, but also in overall structural development (i.e., regional volume) of two of the brain regions we examined. The PCX and DG both exhibited increases in regional volume following DEP exposure only in TLR4^+/-^ fetuses. The persistent effect of prenatal DEP exposure on PCX volume in particular – i.e., the increased volume early in life and decreased volume later in life - is also reflective of the literature on autism. Many clinical studies have investigated aberrancies in brain structure and development from early childhood through adolescence, which is thought to begin in mice around P30 (Spear and Brake, 1983). In early childhood, prior to adolescence, autistic individuals are reported to have significantly larger brain region volumes than their peers (Herbert, 2005). This suggests either excessive neurogenesis (Courchesne et al., 2011) or, perhaps, aberrant apoptosis or phagocytosis by microglia or other cells. Notably, PCX-specific defects have been observed in autistic individuals, both in terms of brain structure and behavior (Courchesne et al., 1993; Egaas et al., 1995). Our study may be one of the first to point toward the biological underpinnings of this association.

Given that males exposed to DEP have a smaller PCX volume, it is logical to assume there might be more microglia-neuron interactions based purely on space constraints within the parenchyma. To address this, we assessed overlap volume in two different ways: total overlap volume, which includes overlaps that occur anywhere on a given microglial cell, as well as cell body overlap, which is restricted to the cell bodies of a chosen microglia-neuron pair and not likely to be impacted by brain region volume. The first measure allows us to ask whether a given microglial cell is more prone to overlaps, including interactions outside of the chosen microglia-neuron pair. The second measure of cell body volumes is specific to each microglia-neuron interaction in question and enables us to compare the degree of specific cell body overlaps between groups. We found that Sex and DEP influenced both measures in the same direction, indicating that microglia in males exposed to DEP had increased overlaps over the entire cell, and that the cell body overlapped significantly more with the neuron of interest, compared to all other groups. If space constraints were the driving force behind such interactions, we would expect to see an increase in overlaps between microglial processes, which survey the parenchyma, and neurons. However, the finding of increased atypical interactions, i.e., cell-body overlaps, suggests a more directed interaction whereby microglial cell bodies are being actively drawn to neuronal cell bodies.

These data are striking because most reported contact between microglia and neurons in the literature is restricted to contact between microglial processes and neuronal synapses. The few instances of published microglia-neuron cell body-to-cell body contact associate such interactions with cellular fusion (Alvarez-Dolado et al., 2003; Ackman et al., 2006; Cusulin et al., 2012), facial nerve axotomy (Blinzinger and Kreutzberg, 1968; Graeber et al., 1988; Kalla et al., 2001; Trapp et al., 2007), neuroprotective synaptic stripping (Raivich et al., 1991; Chen et al., 2014), or phagocytosis of neurons and neuronal precursors (Ribak et al., 2009; Cunningham et al., 2013). The overlaps we quantified in our study are unlikely to be microglia-neuron cellular fusions because this phenomenon is reported to result in hybrid cells that co-express nuclear markers of both cell types and we did not find a single cell nucleus positive for both Iba1 and NeuN. Furthermore, to our knowledge, fusion events involving microglia and neurons have only been reported *in vitro* (Alvarez-Dolado et al., 2003; Ackman et al., 2006), or require the presence of neural stem cells *in vivo* (Cusulin et al., 2012) and may therefore not occur in the intact brain or in the absence of transplantation.

Recently, it was discovered that microglia activated by a LPS preconditioning regimen closely appose neuronal cell bodies and physically displace inhibitory synapses on neurons. This apposition increased neuronal activity and upregulated the expression of antiapoptotic/neurotrophic molecules (Chen et al., 2014). While a protective role for microglia-neuron overlaps could be occurring in our model (**Figure 3B**), this prior study found that maximal microglia-neuron apposition occurred just 24 h after LPS treatment and were transient, nearly disappearing after 2 weeks (Chen et al., 2014). Comparatively, we detect multiple overlaps in our animals over a month after DEP exposure has ended. Finally, no cellular components of neurons were internalized by microglia in their study, whereas we find that significant volumes of neurons are overlapping/within microglia.

The timing of our DEP exposure encompasses developmental neurogenesis. During this period, the number of neural precursor cells that will eventually give rise to cortical neurons is greatly amplified and then reduced to generate the correct number of neurons in the adult mouse (Noctor et al., 2004, 2008). Microglia contribute to this process by reducing the precursor pool in the prenatal brain (Cunningham et al., 2013). If microglia are unable to decrease the precursor pool because of inflammatory activation, and/or a developmental delay, this could result in an excess of neurons, a result that is consistent with our finding that DEP-exposed males have more amoeboid microglia and larger cortical volumes at E18. However, it remains unclear why the increased PCX volume at E18 switched to a decrease at P30. Prior to and leading up to birth, neurons make up most of the cells in the brain (Bandeira et al., 2009). However, by P30, neuron number is no longer the driving force behind cortical volume as neuron numbers have stabilized, peak astrogenesis occurs around P16, and white matter maturation is approximately at adult levels by P24 (Lyck et al., 2007). These data suggest non-neuronal factors such as other glial cells (e.g., astrocytes and/or oligodendrocytes), white matter maturation, or inflammation-induced edema, also play important roles in the persistent modulation of cortical volume beyond early developmental insults, which remains to be explored in our model.

Taken together, our results demonstrate that DEP-exposed male microglia are actively engaged with more neurons, but also that the volume of phagocytic contact throughout the entire cell is greater, suggesting that the microglia-neuron overlaps reflect microglia that are actively phagocytosing (or impaired in their attempt to phagocytose) neurons. Notably, autistic brains have also been found to possess abnormal microglial-neuronal spatial organization, and specifically microglia and neurons were closer together in prefrontal cortex than in controls (Morgan et al., 2010). The shift we observed in microglia of DEP-exposed male offspring toward a more amoeboid morphology, though classically considered to represent a state of higher activation in the adult, may also reflect a more developmentally immature state. Though we did not directly assess maturation in this study, we have recently reported that transcriptional maturation over normal development in the same strain of mice is very closely linked to changes in morphology; that is, a more “activated” phenotype is reflective of a less mature cell, and notably an inflammatory challenge of LPS during development affects male microglial maturation but not female maturation, consistent with the current study (Hanamsagar et al., 2017). Abnormally activated or immature microglia may either exhibit a functional shift toward inflammation/host defense and away from homeostatic phagocytosis, or simply be less efficient at phagocytosing excess neural cells during development, hypotheses that remain to be tested in future studies.

## Author Contributions

JB, SM, RA, and SB designed the experiments; JB, SM, TH, DN, DL, CB, and SM acquired, analyzed, and interpreted the data; JB, SM, and SB wrote the paper.

## Conflict of Interest Statement

The authors declare that the research was conducted in the absence of any commercial or financial relationships that could be construed as a potential conflict of interest.
