# Supplementary material for: Gestational Exposure to Air Pollution Alters Cortical Volume, Microglial Morphology, and Microglia-Neuron Interactions in a Sex-Specific Manner
Source: Front Synaptic Neurosci. 2017 May 31;9:10. doi: 10.3389/fnsyn.2017.00010 (PMC5449437; doi:10.3389/fnsyn.2017.00010)
Supplement: Supplementary file 1 [file Data_Sheet_1.docx]

Supplementary Material

Gestational Exposure to Air Pollution Alters Cortical Volume, Microglial Morphology, and Microglia-Neuron Interactions in a Sex-Specific Manner

Jessica L. Bolton^1,#^, Steven Marinero^2,#^, Tania Hassanzadeh^1^, Divya Natesan^1^, Dominic Le^1^, Christine Belliveau^1^, S. Nicholas Mason^3^, Richard L. Auten^3^, & Staci D. Bilbo^1,2,4^*

^1^Department of Psychology and Neuroscience, Duke University, Durham, NC

^2^Department of Neurobiology, Duke University Medical Center, Durham, NC

^3^Department of Pediatrics, Division of Neonatal Medicine, Duke University Medical Center, Durham, NC

^4^Department of Pediatrics and Program in Neuroscience, Lurie Center for Autism, Harvard Medical School, Massachusetts General Hospital for Children, Boston, MA.

^#^Authors contributed equally to this work

*Correspondence: Staci D. Bilbo: [sbilbo@mgh.harvard.edu](mailto:sbilbo@mgh.harvard.edu)

## Supplementary Figures

**Supplementary Figure 1.** (**A-B**) Example contours drawn around the E18 hippocampus (**A**) and parietal cortex (**B)** in the StereoInvestigator software. Hippocampal regions going clockwise, starting from the bottom left: CA3, CA1, dentate gyrus. (**C**) Example contours drawn around the P30 parietal cortex (top) and dentate gyrus (bottom) in the StereoInvestigator software. Photos were taken of Iba1-stained section at the 4X objective.

**Supplementary Figure 2.** (**A**) No significant differences were detected in CA3 volume at E18. Data are mean ± SEM, (**B**) DEP TLR4+/- males have significantly more stout microglia in the CA3 at E18 than VEH TLR4+/- males, whereas DEP females have significantly fewer stout microglia overall than VEH females. (**C**) No significant differences were detected in CA1 volume at E18. (**D**) DEP TLR4+/- males have significantly more stout microglia in the CA1 at E18 than DEP TLR4-/- males, whereas VEH TLR4+/- females have significantly more stout microglia than DEP TLR4+/- females. Data are mean ± SEM. For A and C, *n*= 6-10/group (note that sexes are combined here). For B and D, *n*= 3-5/group/sex. For B, **p*<0.05 vs. VEH TLR4+/-; ***p*<0.05, DEP vs. VEH. For D, **p*<0.05 vs. DEP TLR4-/- for males; **p*<0.05 vs. DEP TLR4+/- for females.

**Supplementary Figure 3.** Impact of microglia volume on microglia-neuron interactions. To examine the influence of microglia volume on microglia-neuron overlaps, overlap was normalized to whole microglia volume (**B,D**), which included microglia cell bodies and processes. Overlap was also normalized to microglia cell body volume (**C,E**), which excluded the volume of microglial processes. Overlap was measured in two ways: total overlap and cell body overlap. During reconstruction, the microglia and neuron for a given microglia-neuron interaction were isolated by removing microglia and neurons not involved in the cell body-cell body interaction. Within the microglia of interest, overlaps existed both between the cell body of the microglia and neuron, and between microglial processes and surrounding neurons. Total overlap included overlaps throughout the entire microglia and may therefore include overlaps with multiple neurons while cell body overlap is limited to overlapping material within the cell bodies of the selected microglia and neuron. (**A**) DEP-exposed animals have significantly larger microglial cell body volumes than VEH animals. (**B**) Analysis of total overlap normalized to whole microglia volume revealed a significant Sex x DEP interaction which was due to larger overlap volume in DEP males vs. VEH males. (**C**) A very similar pattern was found for total overlap normalized to microglial cell body volume (trend for interaction, although this data failed the normality test). (**D**) Analysis of cell body overlap normalized to whole microglia volume revealed a significant Sex x DEP interaction, which was due to larger overlap volume in DEP M vs. VEH males, as well as in in VEH females vs. VEH males. (**E**) A very similar pattern was found for cell body overlap normalized to microglial cell body volume. Bar graphs represent mean values ± SEM (error bars); n=3 mice/group, 3-5 microglia-neuron renderings per mouse. The data were log-transformed for analysis due to unequal variance but are displayed as raw data in the figures. **p<0.05, DEP vs. VEH; *p < 0.05 vs. VEH male.
